# Integrating Transcriptomic and ChIP-Seq Reveals Important Regulatory Regions Modulating Gene Expression in Myometrium during Implantation in Pigs

**DOI:** 10.3390/biom13010045

**Published:** 2022-12-26

**Authors:** Weiwei Wang, Caiqin Cao, Botao Zhang, Feiyu Wang, Dadong Deng, Jianhua Cao, Hua Li, Mei Yu

**Affiliations:** 1 Key Lab of Agricultural Animal Genetics, Breeding and Reproduction of Ministry of Education, College of Animal Science and Technology, Huazhong Agricultural University, Wuhan 430070, China; 2 Guangdong Provincial Key Laboratory of Animal Molecular Design and Precise Breeding, Foshan University, Foshan 528225, China

**Keywords:** pigs, implantation, myometrium, regulatory regions, H3K27ac, H3K4me3

## Abstract

The myometrium is the outer layer of the uterus. Its contraction and steroidogenic activities are required for embryo implantation. However, the molecular mechanisms underlying its functions remain unknown in pigs. The myometrium includes the inner circular muscle (CM) and the outer longitudinal muscle (LM) layers. In this study, we collected the CM and LM samples from the mesometrial side (named M) of the uterus on days 12 (pre-implantation stage) and 15 (implantation stage) of pregnancy and day 15 of the estrous cycle. The transcriptomic results revealed distinct differences between the uterine CM and LM layers in early pregnancy: the genes expressed in the LM layer were mainly related to contraction pathways, whereas the transcriptional signatures in the CM layer on day 15 of pregnancy were primarily involved in the immune response processes. Subsequent comparisons in the CM layer between pregnant and cyclic gilts show that the transcriptional signatures of the CM layer are implantation-dependent. Next, we investigated the genome-wide profiling of histone H3 lysine 27 acetylation (H3K27ac) and histone H3 lysine 4 trimethylation (H3K4me3) in pig uterine CM and LM layers. The genomic regions that had transcriptional activity and were associated with the expression of genes in the two layers were characterized. Taken together, the regulatory regions identified in the study may contribute to modulating the gene expression in pig uterine CM and LM layers during implantation.

## 1. Introduction

Litter size is one of the most economic traits in swine production. Multiple factors and processes determine the pregnancy outcome. Success in implantation and embryo development is required to achieve a large litter size. The uterus plays a fundamental role in implantation and embryo development during pregnancy. Structurally, the uterus consists of three layers: the endometrium, myometrium, and perimetrium [1]. An endometrium is where the embryo implants and resides during pregnancy. Thus, its role in embryo implantation has been studied intensively and well-determined [2,3,4,5,6]. The myometrium consists of two layers of smooth muscle. Its primary function is to generate uterine contraction and steroidogenic activity, which are also required for implantation [7,8,9].

Much progress has been made toward understanding the physiological mechanisms of myometrial activity in implantation. In polytocous species such as rabbits, mice, rats, and pigs, it is ideal that embryos can implant evenly along the uterine horns. Thus, intrauterine embryo spacing is a common event during pre-implantation. The mechanical force generated by the myometrial activity of the uterus is essential in the migration of embryos in the uterus [9,10,11]. In addition, the myometrium also plays an endocrine role during implantation. Many studies have demonstrated that the pig myometrium could produce prostaglandins, steroid hormones (such as androgens and estrone), and insulin-like growth factor-1 (IGF-1). Moreover, multiple factors, such as interleukin-1β (IL1β), were found to regulate the endocrine role of the myometrium [12,13,14,15]. On the other hand, there have been advances toward understanding the molecular background of myometrial activity. Several studies investigated the transcriptomic profiles of pig myometrium during implantation. Many genes involved in contracting, steroid and prostaglandins synthesis, innate immune response, and immunomodulation were highly expressed in the myometrium [7,15,16,17]. The findings indicate that, in addition to the contracting activity, pig myometrium has an essential role in creating an optimal environment for embryo implantation.

The myometrium includes the inner circular muscle (CM) layer and the outer longitudinal muscle (LM) layer [18,19,20]. The CM layer is exclusively innervated by cholinergic nerves, whereas the LM layer is mainly innervated by adrenergic nerves [21,22,23,24,25]. In addition, the responsiveness of the CM and LM layers to bioactive substances is different. For example, the CM layer is more sensitive to 5-hydroxytryptamine [26] than the LM layer, while the LM layer is more sensitive to acetylcholine [27], norepinephrine [23], and histamine [28] than the CM layer. Thus, the role of the CM and LM layers may differ during implantation. Indeed, it has been reported that vascular smooth muscle cells (vSMCs) contain contractile and synthetic phenotypes that differ in marker expression, morphology, and activity [29,30]. Furthermore, switching from a contractile to synthetic vSMC phenotype can respond to various physiological changes [31,32,33]. Therefore, investigating the transcriptional regulation of genes in uterine CM and LM tissues will provide further evidence for exploring the molecular mechanism of the role of the myometrium in embryo implantation.

Gene regulation involves multiple factors, such as transcription factors, cofactors, non-coding RNAs, and histone modifications [34,35]. Histone modifications, such as methylation and acetylation, play a critical role in regulating gene expression by affecting the chromatin structure and modifying the access of transcription factors and RNA polymerases to gene promoters [36]. H3K4me3 and H3K27ac are the two well-studied histone modifications. H3K4me3 is enriched in the promoter region, while H3K27ac marks the proximal and distal regions of the transcription start site (TSS). Recent studies revealed that H3K4me3 and H3K27ac could regulate various biological processes, such as embryonic development [37,38,39,40,41,42]. Our previous study has identified the genomic localization of H3K4me3 and H3K27ac in pig placentas by chromatin immunoprecipitation sequencing (ChIP-seq) [43]. Recently, many studies investigated the architecture of chromatin in multiple tissues from different pig breeds and characterized the function of the *cis*-regulatory elements marked by H3K4me3 and H3K27ac in the pig genome [44,45,46,47,48]. A recent study revealed that the myometrium undergoes a cascade of epigenetic events during active labor and postpartum in the mouse model, indicating that histone modifications contribute to regulating gene expression in the myometrium [49]. However, little is known about the chromatin landscape on a genome-wide scale in pig myometrium during implantation.

In the present study, we collected the myometrial samples (including the CM and LM layers) from pregnant pigs (days 12 and 15) and cyclic pigs (day 15) and carried out the RNA-seq and ChIP-seq. For the first time, this study (1) characterized the transcriptional signatures in pig uterine CM and LM layers during implantation, and (2) identified the regulatory regions important to gene expression in the two layers during implantation. The data will provide valuable resources for understanding the mechanisms involved in implantation and identifying functional variants in the pig genome.

## 2. Materials and Methods

### 2.1. Sample Collection

All animal procedures were performed according to the protocols approved by the Huazhong Agricultural University Ethics Committee (HZAUSW-2016-015). The estrus behavior of Yorkshire gilts was observed using healthy boar. Then, (1) uterine samples were collected from the gilts on day 15 of the estrous cycle (*n* = 3 gilts), and (2) the rest of the gilts were naturally mated with boar at the onset of the second estrus (Day 0), and again 12 h later, and uterine samples were collected on days 12 and 15 of pregnancy (*n* = 3 gilts/day). The uterus was quickly placed on ice and cut into 10–15 cm segments. The uterine segments were flushed with cold RNase-free phosphate-buffered saline (PBS), and pregnancy was confirmed by the presence of conceptuses in uterine flushing. The uterine samples not flushed with PBS were embedded in precooled optimum cutting temperature (OCT) compound (SAKURA Tissue-Tek, Torrance, CA, USA), snap-frozen in liquid nitrogen, and stored at −80 °C. Next, the endometrium from the mesometrial (named M) side was removed with sterile scalpel blades at −30 °C. Subsequently, the circular muscles (named CM) and longitudinal muscles (named LM) were carefully separated and collected, respectively. All samples were stored at −80 °C.

### 2.2. RNA Library Construction and RNA-Seq Data Analysis

The uterine circular muscles (CM) and longitudinal muscles (LM) were collected from the mesometrial side (named M). Six types of pig myometrium samples were used for RNA-seq (*n* = 3 gilts each). The sample types included: (1) CM and LM collected from the mesometrial side of the uterus on day 12 of pregnancy (named GD12M_CM and GD12M_LM, respectively), (2) CM and LM collected from the mesometrial side of the uterus on day 15 of pregnancy (named GD15M_CM and GD15M_LM, respectively), and (3) CM and LM collected from the mesometrial side of the uterus on day 15 of the estrous cycle (named CD15M_CM and CD15M_LM, respectively). Total RNA was isolated using the HP Total RNA Kit (R6812, Omega Bio-tek, Norcross, GA, USA) according to the manufacturer’s instructions. The quality, concentration, and integrity of the total RNA were determined using the NanoDrop spectrophotometer (BIO-DL, Shanghai, China), Qubit 2.0 Fluorometer (Invitrogen, Carlsbad, CA, USA), and Bioanalyzer 2100 system (Agilent, Santa Clara, CA, USA), respectively. A total of 18 libraries (*n* = 3 gilts per sample type) were constructed for RNA sequencing using the Illumina HiSeq X Ten (PE150) platform (Novogene, Beijing, China). The reads were mapped to the *Sus scrofa* genome assembly 11.1 (ENSEMBLE: ftp://ftp.ensembl.org/pub/release-93/gtf (accessed on 24 June 2021)) with HISAT2 (v2.1.0) [50]. The mapped reads were assembled by HTSeq (v0.9.1) [51]. Information on the quality of the RNA-seq data is listed in Table S1. Hierarchical clustering and principal component analysis of the annotated genes were performed using R (v4.0.5). Differentially expressed genes (DEGs) were identified using DESeq2 [52]. The DEGs between the uterine CM and LM were analyzed using the paired design. In contrast, the DEGs between days 15 and 12 of pregnancy and between day 15 of pregnancy and day 15 of the estrous cycle were analyzed using the unpaired design, respectively. The Benjamini–Hochberg method was used to determine the false discovery rate (FDR)-adjusted *p*-values. Genes with adjusted *p*-value < 0.05, |log_2_foldchange| > 1 and base mean > 100 were identified as DEGs. The Venn analysis of the DEGs was performed using Venny (v2.1.0) (https://bioinfogp.cnb.csic.es/tools/venny/index.html (accessed on 14 December 2022)). Gene ontology (GO) analysis for the DEGs was carried out using DAVID Bioinformatics Resources (2021 Update) (https://david.ncifcrf.gov/ (accessed on 16 May 2022)) [53].

### 2.3. Quantitative Real-Time PCR

Quantitative real-time PCR (qRT-PCR) was carried out to validate the RNA-seq data in the same RNA samples used for RNA sequencing. The total RNA was reverse-transcribed into cDNA using FastKing gDNA Dispelling RT SuperMix (Tiangen Biotech, Beijing, China) according to the manufacturer’s instructions. The gene-specific primers are listed in Table S1. The glyceraldehyde 3-phosphate dehydrogenase (GAPDH) gene was used as a control [15]. The qRT-PCR was performed using Hieff UNICON^®^ qPCR SYBR Green Master Mix (Yeasen, Shanghai, China) in a Bio-Rad CFX384 Touch Real-Time PCR Detection System (Bio-Rad Laboratories, Hercules, CA, USA). The paired or unpaired Student’s *t*-test in R (v4.0.5) was used for statistical analysis. A *p*-value of < 0.05 was considered statistically significant.

### 2.4. ChIP-Seq Library Construction and Sequencing

To investigate the regulatory mechanisms underlying the pregnancy-dependent transcriptional changes on day 15, we examined the genome-wide profiling of H3K27ac and H3K4me3 in three types of myometrium samples (GD15M_CM, GD15M_LM, and CD15M_CM). The samples (*n* = 2 gilts for each type) were ground to a fine powder in liquid nitrogen and processed using the Cleavage Under Targets and Tagmentation (CUT&Tag) method [44,54] with minor modifications. The ChIP-grade antibodies H3K4me3 (ab8580, Abcam, Cambridge, UK) and H3K27ac (ab4729, Abcam, Cambridge, UK) were used in this study. The samples were lysed with precooled lysis buffer at 4 °C for 10 min, and resuspended in wash buffer (150 mM NaCl, 30 mM HEPES (pH 7.5), 0.5 mM spermidine, and protease inhibitor). Then, the nuclei were counted. Next, 10 µL Concanavalin A beads were mixed in 100 µL binding buffer (10 mM KCl, 1 mM MnCl_2_, 30 mM HEPES (pH 7.5), and 1 mM CaCl_2_). After discarding the supernatant, 10 µL binding buffer was re-added to equilibrate the beads. Approximately 100,000 nuclei were resuspended in wash buffer, mixed with equilibrated Concanavalin A beads, rotated, and incubated for 15 min at room temperature. Then, the beads were resuspended in a mixture containing 0.05% digitonin, 1.5 mM EDTA, and a 1:50 diluted primary antibody, rotated, and incubated for 3 h at room temperature. After incubation, the beads were washed three times with Dig-wash buffer (150 mM NaCl, 30 mM HEPES (pH 7.5), 0.5 mM spermidine, 0.05% digitonin, and protease inhibitor), and incubated with pA-Tn5 (1:100) in 100 µL Dig-300 buffer (450 mM NaCl, 30 mM HEPES (pH 7.5), 0.5 mM spermidine, 0.01% digitonin, and protease inhibitor) for 1 h at room temperature. Next, the beads were washed three times with Dig-300 buffer, resuspended in 100 µL tagmentation buffer (1 mM MgCl_2_ and Dig-300 buffer), and incubated on a ThermoMixer at 37 °C for 1 h. After discarding the supernatant, the beads were suspended in 150 µL elution buffer and incubated on a ThermoMixer at 65 °C for 1 h. Similarly, after discarding the supernatant, 250 µL TE buffer and protease K were added to the beads and incubated on a ThermoMixer at 55 °C for 6 h. After incubation, 400 µL saturated phenol was added to the supernatant, mixed well, and centrifuged at 15,000 rpm for 5 min at room temperature. After this, 5 M NaCl, glycogen, and precooled 100% ethanol were sequentially added to the supernatant to precipitate the DNA, eluted with precooled 75% ethanol and solubilized with 30 µL ddH_2_O. The immunoprecipitated DNA and immunoprecipitated control (IgG) were purified. ChIP-seq libraries were constructed following the manufacturer’s instructions for Illumina, and were sequenced on an Illumina NovaSeq PE150 platform (Yingzi Gene Technology, Wuhan, China).

### 2.5. ChIP-Seq Data Analysis

The clean data were mapped to the *Sus scrofa* genome assembly 11.1 with BWA (v0.7.17-r1188) [55]. The duplicate reads and low-quality reads (MAPQ < 25) were filtered out using SAMtools (v1.9) [56] and Picard (v2.8.3). Information about the number and quality of reads in ChIP-seq data is listed in Table S1. To identify the H3K4me3 and H3K27ac peaks, replicates of ChIP-seq data were pooled and the peak calling was performed by MACS2 (v2.2.6) [57] with 3 kb extension regions. The modifications of H3K4me3 and H3K27ac were normalized with IgG files as controls using bamCompare of deepTools (v3.5.1) [58]. The enrichment profiles were performed using computeMatrix and plotHeatmap of deepTools. The annotation of modification regions of H3K4me3 and H3K27ac were achieved using ChIPpeakAnno (v3.0.0) [59] and ChIPseeker (v1.26.2) [60]. The modification regions of H3K4me3 and H3K27ac in the uterine CM and LM layers were differentially analyzed using DiffBind (v3.4) [61], and regions with the false discovery rate (FDR) < 0.05 were defined as differential modification regions.

### 2.6. Dual-Luciferase Reporter Assays

The binding sites of transcription factors in the differential histone modification regions within the TSS ± 1 kb region of the DEGs were predicted by the JASPAR database (2022) (https://jaspar.genereg.net/ (accessed on 10 August 2022)) [62]. A dual-luciferase reporter assay was used to validate the promoter activity. The promoter sequences of the DEGs were cloned into the pGL3-Basic vector (Promega, Madison, WI, USA) using the restriction sites of HindIII and XhoI (NEB, Beijing, China) to construct target gene vectors. All constructed vectors were verified by DNA sequencing. The primers for the dual-luciferase reporter assay are listed in Table S1. Vectors were co-transfected with PK15 cells using Lipofectamine 2000 (Invitrogen, Carlsbad, CA, USA), with the pRL-TK (Promega, Madison, WI, USA) and pGL3-Basic vectors as internal and negative controls, respectively. Then, 24 h after transfection, the cells were collected and detected using the Dual-Luciferase Reporter Gene Assay Kit (Yeasen, Shanghai, China). Each target gene vector was assayed with three biological replicates and at least four technical replicates. A *p*-value < 0.05 for the Student’s *t*-test was considered significant.

## 3. Results

### 3.1. Distinct Transcriptional Signatures in Pig Uterine Circular and Longitudinal Muscles during Implantation

**Figure 1:**
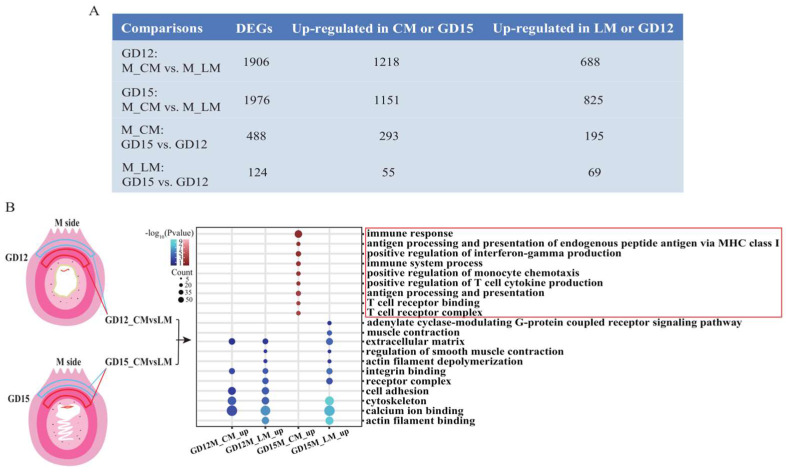
Transcriptional signatures in the M_CM and M_LM on GD12 and GD15 in pigs. (**A**) Identification of DEGs between M_CM and M_LM on GD12 and GD15. (**B**) GO analysis of DEGs between M_CM and M_LM on GD12 and GD15. GD12, day 12 of pregnancy; GD15, day 15 of pregnancy; M, the mesometrial side of the uterus; CM, the circular muscle of myometrium; LM, the longitudinal muscle of myometrium; DEGs, differentially expressed genes.

The transcriptome data were obtained from four types of myometrium samples (GD12M_CM, GD12M_LM, GD15M_CM, and GD15M_LM) (Table S1). The hierarchical clustering and principal component analyses revealed the discrimination among the four types based on the transcriptome data (Figure S1A,B). Next, we identified the differentially expressed genes (DEGs) from four comparisons, of which two were for identifying the DEGs between the two layers (GD12M_CM vs. GD12M_LM; GD15M_CM vs. GD15M_LM), and the other two were for identifying the DEGs between the days of pregnancy (GD15M_CM vs. GD12M_CM; GD15M_LM vs. GD12M_LM) (Table S2). The results showed that the number of DEGs between the CM and LM layers on days 12 and 15 of pregnancy was much more than that of DEGs between the days of pregnancy (Figure 1A and Figure S1C,D).

Therefore, the GO analysis was restricted to the DEGs identified between the two layers on days 12 and 15 of pregnancy. Briefly, on day 12 of pregnancy, in addition to the common pathways enriched in the genes upregulated in the two layers, smooth muscle contraction pathways were mainly enriched for genes upregulated in the LM layer other than the CM layer (Figure 1B). On the other hand, on day 15 of pregnancy, the upregulated genes in the LM layer were enriched in terms similar to those enriched for DEGs identified between the two layers on day 12 of pregnancy, but the upregulated genes in the CM layer were significantly associated with immune response, the positive regulation of interferon-gamma production, antigen processing and presentation, and T cell receptor binding (Figure 1B; Table S2). Taken together, we found that (1) most of the upregulated genes in the LM layer were related to the smooth muscle contraction pathways, and (2) the transcriptional signatures of the CM layer changed from day 12 to day 15 of pregnancy and became distinctly different from those of the LM layer on day 15 of pregnancy.

### 3.2. Transcriptional Signatures of GD15M_CM Were Pregnancy-Dependent and Distinct from CD15M_CM

**Figure 2:**
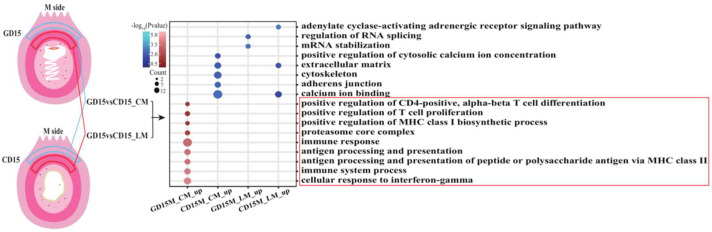
Differences in the transcriptional signatures in M_CM and M_LM between GD15 and CD15 in pigs. GD15, day 15 of pregnancy; CD15, day 15 of the estrous cycle; M, the mesometrial side of the uterus; CM, the circular muscle of myometrium; LM, the longitudinal muscle of myometrium; DEGs, differentially expressed genes.

We next investigated whether the transcriptional signatures in GD15M_CM were associated with conceptus implantation. The analyses were performed by integrating the transcriptional data of the uterine smooth muscles (including LM and CM) from the M side of the uterus on day 15 of pregnancy and day 15 of the estrous cycle. The hierarchical clustering and principal component analysis indicated the discrimination among the different types based on the transcriptome data (Figure S2A,B). Subsequent analyses found that 385 and 143 DEGs were identified from the comparisons of GD15M_CM vs. CD15M_CM and GD15M_LM vs. CD15M_LM, respectively (Table S2). Figure 2 shows a distinct difference in the enriched GO terms between GD15M_CM and CD15M_CM. The upregulated genes in GD15M_CM were mainly related to immune response processes; however, the upregulated genes in CD15M_CM were associated with calcium ion binding, cytoskeleton, and extracellular matrix (Table S2). The findings suggest that transcriptional signatures in the CM layer on day 15 of pregnancy might be pregnancy-dependent.

### 3.3. Validation of the Expression Patterns of the Differentially Expressed Genes

**Figure 3:**
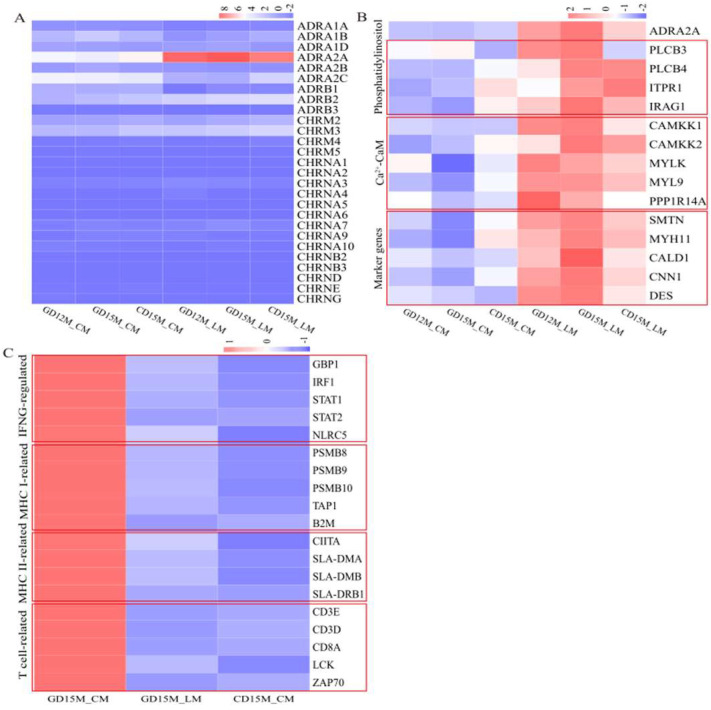
Heatmaps of the DEGs. (**A**) Heatmap of adrenergic and cholinergic receptor genes in M_CM and M_LM on GD12, GD15, and CD15. (**B**) Heatmap of genes in the phosphatidylinositol and Ca^2+^–CaM signaling pathways in M_CM and M_LM on GD12, GD15, and CD15. (**C**) Heatmap of immune response processes–related genes in M_CM and M_LM on GD15 and M_CM on CD15. GD12, day 12 of pregnancy; GD15, day 15 of pregnancy; CD15, day 15 of the estrous cycle; M, the mesometrial side of the uterus; CM, the circular muscle of myometrium; LM, the longitudinal muscle of myometrium; DEGs, differentially expressed genes.

Our RNA-seq data shows that *ADRA2A* (Adrenoceptor alpha 2A), one of the 9 adrenergic and 18 cholinergic receptor genes, was highly expressed in the LM layer (Figure 3A). *ADRA2A* is a G protein-coupled receptor that regulates smooth muscle contraction through the phosphatidylinositol signaling pathway [63,64]. As expected, the expression of some genes in the phosphatidylinositol and Ca^2+^-CaM signaling pathways was detected in our RNA-seq data (Figure 3B). The genes included *PLCB3* (Phospholipase C beta 3), *PLCB4* (Phospholipase C beta 4), *ITPR1* (Inositol 1,4,5-trisphosphate receptor type 1), *IRAG1* (Inositol 1,4,5-triphosphate receptor associated 1), *CAMKK1* (Calcium/calmodulin dependent protein kinase kinase 1), *MYL9* (Myosin light chain 9), and *PPP1R14A* (Protein phosphatase 1 regulatory inhibitor subunit 14A). Consistently, the qRT-PCR confirmed the expression patterns of the genes listed above, and also the genes of *MYH11* (Myosin heavy chain 11) and *SMTN* (Smoothelin), two smooth muscle contraction marker genes [65,66] (Figure S3A,B).

On the other hand, our RNA-seq data revealed that the expressions of genes in the cellular response to interferon-gamma and the antigen processing and presentation pathways were highly expressed in the GD15M_CM (Figure 3C). The genes included: (1) IFNG-regulated genes, including *GBP1* (Guanylate binding protein 1), *IRF1* (Interferon regulatory factor 1), *STAT1* (Signal transducer and activator of transcription 1), *STAT2* (Signal transducer and activator of transcription 2), and *NLRC5* (NLR family CARD domain containing 5); (2) Major histocompatibility complex (MHC) class I-related genes, including *PSMB8* (Proteasome 20S subunit beta 8), *PSMB9* (Proteasome 20S subunit beta 9), *PSMB10* (Proteasome 20S subunit beta 10), *TAP1* (Transporter 1, ATP binding cassette subfamily B member), and *B2M* (Beta-2-microglobulin); (3) MHC class II-related genes, including *CIITA* (Class II major histocompatibility complex transactivator), *SLA-DMA* (SLA-DM alpha chain), *SLA-DMB* (MHC class II, DM beta), and *SLA-DRB1* (MHC class II histocompatibility antigen SLA-DRB1); and (4) T cell-related genes, including *CD3E* (CD3 epsilon subunit of T cell receptor complex), *CD3D* (CD3 delta subunit of T cell receptor complex), *CD8A* (CD8a molecule), *LCK* (LCK proto-oncogene, Src family tyrosine kinase), and *ZAP70* (Zeta chain of T cell receptor-associated protein kinase 70). Expectedly, the qRT-PCR results confirmed that those genes were expressed at significantly higher levels in the GD15M_CM (Figure S3C,D).

### 3.4. Genome-Wide Maps of H3K4me3 and H3K27ac in Pig Uterine Smooth Muscle

**Figure 4:**
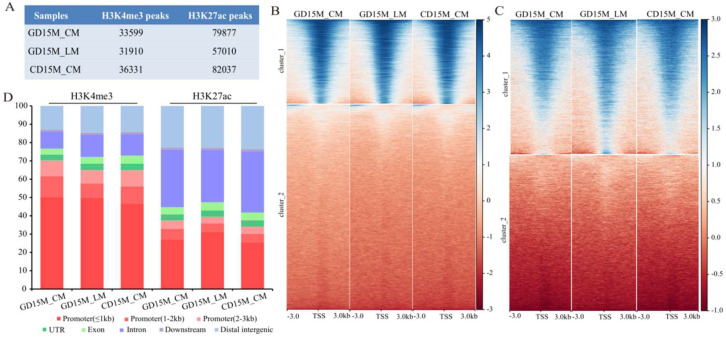
Genome–wide landscape of H3K4me3 and H3K27ac modifications in M_CM and M_LM on GD15 and M_CM on CD15. (**A**) The number of H3K4me3 and H3K27ac peaks identified in M_CM and M_LM. (**B**) Heatmap of the H3K4me3 signals within ±3 kb from the TSS that was developed according to gene expression level. (**C**) Heatmap of the H3K27ac signals within ±3 kb from the TSS that was developed according to gene expression level. (**D**) Distribution of the H3K4me3 and H3K27ac modification regions in genomic regions. GD15, day 15 of pregnancy; CD15, day 15 of the estrous cycle; M, the mesometrial side of the uterus; CM, the circular muscle of myometrium; LM, the longitudinal muscle of myometrium.

ChIP-seq of H3K4me3 and H3K27ac was performed in three types of myometrium samples (GD15M_CM, GD15M_LM, and CD15M_CM) (Figure 4A; Table S3), focusing on investigating the regulatory mechanisms underlying the pregnant-dependent transcriptional changes identified in the uterine CM and LM layers. As shown in Figure 4B–D, the H3K4me3 and H3K27ac signals peaked within ± 3 kb from the transcription start site (TSS), and most modification regions of H3K4me3 were in the promoter region, while most of the modification regions of H3K27ac were in the intron and distal intergenic region. We next compared the differential histone modification regions between GD15M_CM and CD15M_CM and between GD15M_CM and GD15M_LM. A total of 1,637 and 19 differential histone modification regions were identified between GD15M_CM and CD15M_CM for H3K4me3 and H3K27ac, respectively. In addition, 11,538 and 6536 differential histone modification regions were identified between GD15M_CM and GD15M_LM for H3K4me3 and H3K27ac, respectively (FDR < 0.05; Table S4).

### 3.5. Investigating the H3K4me3 and H3K27ac Modified Regions That Regulate the Gene Expression in Longitudinal Muscle

**Figure 5:**
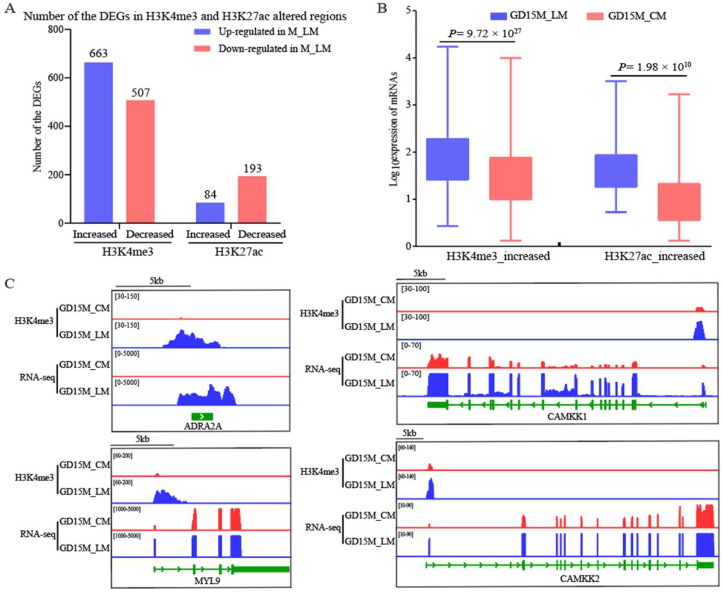
Relationships of H3K4me3 and H3K27ac modifications with the DEGs between the M_LM and M_CM on GD15. (**A**) Number of DEGs containing H3K4me3 or H3K27ac-increased or -decreased regions. (**B**) Box plots of the expression value of the upregulated genes in GD15M_LM that H3K4me3 or H3K27ac modified. (**C**) IGV (Integrative Genome Viewer) views of the H3K4me3 modification patterns. GD15, day 15 of pregnancy; M, the mesometrial side of the uterus; CM, the circular muscle of myometrium; LM, the longitudinal muscle of myometrium; DEGs, differentially expressed genes.

A total of 1439 H3K4me3 and 382 H3K27ac differential peaks within ±3 kb regions from the TSS were detected in 1217 DEGs between the LM and CM layers from pregnant gilts on day 15 (Table S5). Figure 5A shows the number of DEGs containing H3K4me3 or H3K27ac-increased or -decreased regions. Due to our findings that the expression of the genes related to smooth muscle contraction pathways was increased in the LM layer, we further focused on identifying the H3K4me3 or H3K27ac-increased regions that may regulate the expression of these genes. The expression levels of the upregulated genes in GD15M_LM were quantified using RNA-seq data. The results showed that the increase in expression levels of the H3K4me3-increased region containing genes was significantly associated with the rise of H3K4me3 signals (*p* = 9.72 × 10^27^; Figure 5B). Similarly, the increase in the expression levels of the H3K27ac-increased region containing genes was significantly associated with increased H3K27ac signals (*p* = 1.98 × 10^10^; Figure 5B). Specifically, some genes, such as *ADRA2A*, *CAMKK1*, *CAMKK2*, and *MYL9*, showed increased expression and enrichment of H3K4me3 marks (Figure 5C and Figure S4A; Table S5).

### 3.6. Investigating the H3K4me3 and H3K27ac Modified Regions That Regulate the Gene Expression in Circular Muscle

**Figure 6:**
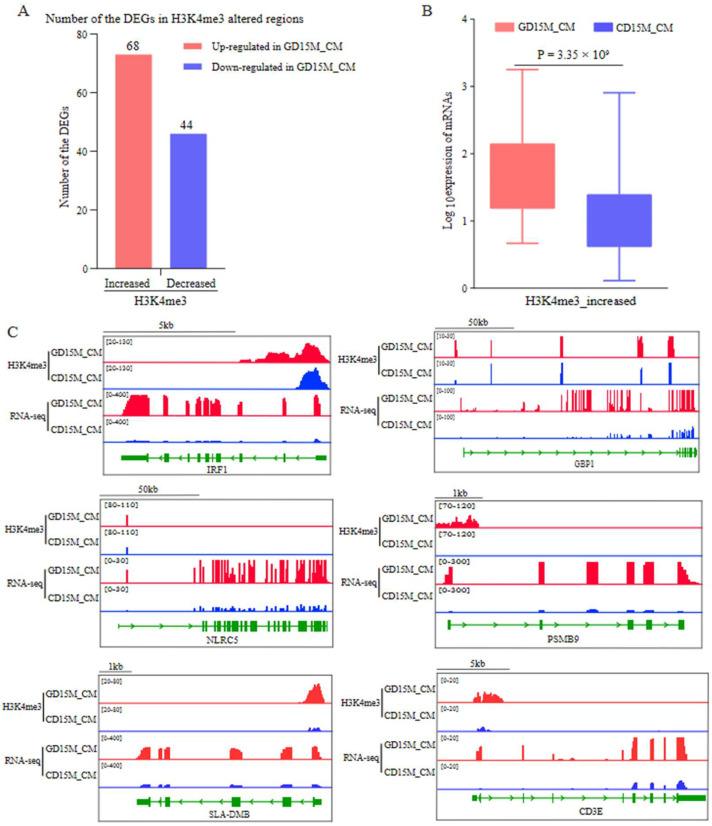
Relationships of H3K4me3 and H3K27ac modifications with the DEGs between the M_CM on GD15 and CD15. (**A**) Number of DEGs containing H3K4me3-increased or -decreased regions. (**B**) Box plots of the expression value of the upregulated genes in GD15M_CM that H3K4me3 modified. (**C**) IGV views of the H3K4me3 modification patterns. GD15, day 15 of pregnancy; CD15, day 15 of the estrous cycle; M, the mesometrial side of the uterus; CM, the circular muscle of myometrium; DEGs, differentially expressed genes.

The differential modification regions within ±3 kb region from the TSS of the DEGs between GD15M_CM and CD15M_CM were analyzed. A total of 125 H3K4me3 and 0 H3K27ac differential peaks were detected in 112 DEGs (Table S5). Of the 112 DEGs, 68 genes upregulated in GD15M_CM had H3K4me3-increased signals (Figure 6A). The expression levels of the genes were quantified using RNA-seq data. As shown in Figure 6B, the increased expression of the genes in GD15M_CM was significantly associated with the enrichment of H3K4me3 marks (*p* = 3.35 × 10^9^). Specifically, many genes, such as *IRF1*, *GBP1*, *NLRC5*, *PSMB9*, *SLA-DMB*, and *CD3E*, showed increased expression and enrichment of H3K4me3 marks (Figure 6C and Figure S4B; Table S5).

### 3.7. Validation of the Promoter Activity of the Identified Regulatory Regions

**Figure 7:**
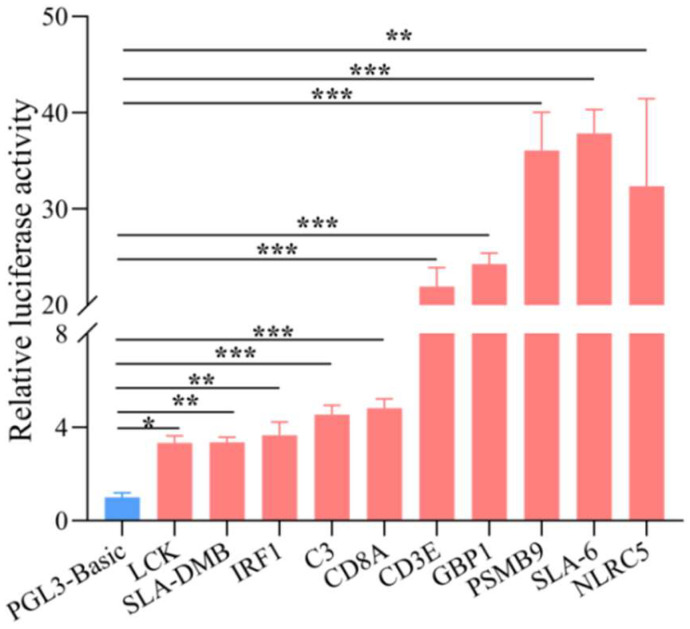
Validation of the promoter activity of immune response processes-related genes by dual-luciferase reporter assays. Data are represented as mean ± SEM, and * *p* < 0.05, ** *p* < 0.01, *** *p* < 0.001.

Furthermore, we examined whether the differential H3K4me3 modification regions within ±1 kb from the TSS had activity to promote the expression of immune response processes-related genes. Dual-luciferase reporter assays were performed with empty vector (pGL3-Basic) and target gene vectors. Figure 7 shows that the activities of immune response processes-related genes, including *LCK*, *SLA-DMB*, *IRF1*, *C3* (Complement C3), *CD8A*, *CD3E*, *GBP1*, *PSMB9*, *SLA-6* (MHC class I antigen 6), and *NLRC5*, were significantly or extremely significantly elevated. Subsequently, we predicted the binding sites of transcription factors in the regions. Table 1 shows that four genes, *IRF1*, *NLRC5*, *GBP1*, and *PSMB9*, have a STAT1 (Signal transducer and activator of transcription 1) binding site, while *GBP1* and *PSMB9* contain the IRF1 (Interferon regulatory factor 1) binding site as well.

## 4. Discussion

Pig embryo implantation is a complex process during which coordinated embryo–uterine interactions are essential. The myometrium, one of the layers of the uterus, is a type of involuntary non-striated muscle. Its contractility helps the embryos enter the uterine horn from the oviductal-uterine junction and evenly distribute and space out along the longitudinal axis before their implantation sites are finally fixed. In addition, the myometrium also has an endocrine function required for establishing implantation [9,10]. Thus, the physiological role the myometrium plays has important implications for embryo survival. This study aimed to investigate the molecular regulatory mechanism of its physiological function. We identified the uterine CM and LM transcriptional profiles from early pregnant pigs (days 12 and 15) and cyclic pigs (day 15). In addition, the genome-scale profiles of H3K4me3 and H3K27ac in the uterine CM and LM from pregnant pigs and CM from cyclic pigs (day 15) were determined. The novel findings of this study include that (1) the transcriptional signature of the uterine CM became different from that of LM on day 15 of pregnancy and is distinguished from that of CM on day 15 of the estrous cycle, and (2) the regulatory regions that modulate gene expression in pig uterine CM and LM during implantation are characterized.

The uterine myometrium consists of two layers: the circular muscle (CM) and longitudinal muscles (LM). Previously, Kurowicka et al. reported that the contractility of the LM layer is significantly higher than that of the CM layer in early pregnant (days 14–16) and cyclic (days 14–16) pigs [67]. In addition, Franczak et al. investigated the alterations in the myometrial transcriptome of early pregnant versus cyclic pigs using the microarray technique and found that, besides playing a role in contracting, the pig myometrium has other functions involved in endocrine and immune signaling [7], but the molecular mechanisms underlying the functions of the two smooth muscle layers are unclear. For the first time, this study determined the uterine CM and LM transcriptional profiles from pregnant pigs (days 12 and 15) and cyclic pigs (day 15). Days 12 and 15 of pregnancy were selected as stages of the maternal recognition of pregnancy and early embryo attachment, respectively [68]. Distinct differences in the transcriptional profiles between the uterine CM and LM were found. Briefly, on day 12 of pregnancy, the differences were mainly caused by the differences in expression levels of the genes related to muscle contraction pathways; however, on day 15 of pregnancy, the expression of the genes related to muscle contraction pathways was maintained in the uterine LM, but the genes involved in immune signaling were upregulated in the CM. In addition, the onset of luteolysis in pigs appears around day 15 of the estrous cycle [69]. Thus, the significant findings of our transcriptional profile analysis include that: (1) the function of the two smooth muscle layers became different on day 15 of pregnancy, the early embryo attachment stage in pigs, and (2) the LM layer maintains the contraction function in pregnant and cyclic pigs (day 15), but the CM layer mainly plays a role in immune response rather than contraction on day 15 of pregnancy. Taken together, we provided evidence at the level of transcription to explain the difference in the physiological roles of the two smooth muscle layers during implantation.

The sympathetic system modulates uterine contraction. Noradrenalin and cholinergic regulate uterine contraction by acting directly through the corresponding receptors in the myometrium during pregnancy [70,71]. This study detected the expression of all 9 adrenergic and 18 cholinergic receptor genes in the myometrium from the early pregnant pigs (days 12 and 15) and cyclic pigs (day 15). It is worth noting that all 27 receptor genes were expressed at a much lower level in the CM layer, whereas the *ADRA2A*, encoding the α2-adrenoceptor subtype, is the predominantly expressed receptor gene in the LM layer. α2-adrenoceptor belongs to the G protein-coupled receptor family, which regulates smooth muscle cell contraction by acting through the phosphatidylinositol signaling pathway to activate the calmodulin signaling pathway [63,64]. Consistently, some genes in the phosphatidylinositol and the calmodulin signaling pathways showed higher expression in the LM layer compared to the CM layer. Moreover, marker genes of smooth muscle contraction (*MYH11* and *SMTN*) were also expressed at a higher level in the LM layer. The findings suggest that the α2-adrenoceptor-mediated signaling may contribute to the contraction function of the LM layer in pregnant and cyclic pigs. Noradrenaline is a natural ligand for α-adrenoceptors. Many studies in model animals have demonstrated that interfering adrenergic signaling during early pregnancy can disrupt embryo distribution along the longitudinal axis, leading to embryo loss [72,73,74]. Therefore, our findings suggest that the α2-adrenoceptor-regulated contraction of the LM layer may have implications for regulating intrauterine embryo distribution and spacing during early pregnancy in pigs.

This study found that genes related to immune response processes were upregulated in the CM layer on day 15 of pregnancy. On the other hand, we provided evidence to show that the transcriptional signature appears at the early embryo attachment stage and is implantation-dependent. The immune response processes identified include the cellular response to interferon-gamma, antigen processing, and presentation. Major histocompatibility complex (MHC) classes I and II are involved in processing endogenous and exogenous antigens and presenting antigens to the antigen-presenting cells [75]. IFN-gamma is a cytokine that participates in the processing and presentation of antigens through class I and class II pathways [76]. Pig conceptuses secrete IFN-gamma between days 12 and 20 of pregnancy and reach peak values around days 15–16 [77]. Studies have shown that IFN-gamma derived from conceptuses can regulate the expression of MHC class I and II genes in pig endometrium, which may create an immune environment in the pig endometrium to protect the mother and permit the implantation of conceptuses [3,78,79,80,81]. Therefore, it seems that similar to the endometrium, a proper immune microenvironment in the CM layer is required for implantation, and the immune system in the CM layer might undergo remodeling induced by conceptus IFN-gamma.

As described above, we found that the LM and CM layers have distinct transcriptional signatures at the early attachment stage, and we examined the gene regulatory mechanisms underlying the difference. The regulation of gene expression is primarily controlled by *cis*-regulatory elements (CREs), including promoters and enhancers, which can be identified with techniques such as ChIP-seq [82]. Our genome-wide ChIP-seq analyses of H3K4me3 or H3K27ac revealed many histone modification regions in the two layers. In addition, we identified the genomic regions in which H3K4me3 or H3K27ac were altered between the CM and LM layers and between pregnant and cyclic pigs on day 15. Furthermore, our data show that changes in the levels of H3K4me3 or H3K27ac at promoters were positively associated with the expression levels of the neighboring genes, indicating the role of H3K4me3 or H3K27ac in regulating gene expressions in the two layers during implantation.

In humans and other animals, a large number of *cis*-regulatory elements have been determined using the ChIP-seq technique. Many *cis*-regulatory elements significantly contribute to phenotypic variation in complex traits [48,83,84]. We then examined the elements that may regulate the differential expression of the *ADRA2A* and some immune response genes identified in the study. A recent study on subjects with schizophrenia characterized that a promoter region in *ADRA2A*, epigenetically modified by both H3K4me3 and H3K27me3, could modulate the expression of *ADRA2A* in the dorsolateral prefrontal cortex [85]. Consistently, this study found that *ADRA2A* was highly expressed in the LM layer, and H3K4me3 was significantly enriched in its proximal promoter (≤1 kb). Thus, the proximal promoter we identified might contribute to modulating the expression of *ADRA2A* in the pig LM layer during implantation and is worthy of further study. Meanwhile, the promoters marked by H3K4me3 were identified in some immune response genes highly expressed in the pig CM layer on day 15 of pregnancy. Moreover, we validated the transcriptional activity of the promoters in several genes, including *LCK*, *SLA-DMB*, *IRF1*, *C3*, *CD8A*, *CD3E*, *GBP1*, *PSMB9*, *SLA-6*, and *NLRC5*. *Cis*-regulatory elements are non-coding DNA regions with transcriptional regulatory activity. A typical *cis*-regulatory element consists of binding sites for transcription factors and other associated cofactors that determine its function [83,86]. *STAT1* is a member of the STAT protein family. It can be activated by various ligands (such as IFN-gamma) and then translocated to the cell nucleus to drive the expression of the target genes. Many target genes of *STAT1* have been identified, including *IRF1*, *GBP1*, *NLRC5*, *PSMB9*, and *CIITA* [79,87,88,89,90]. Consistent with the previous findings, our study found that *STAT1* gene expression was upregulated in the CM layer on day 15 of pregnancy and detected the *STAT1* binding site in the promotor regions identified in *IRF1*, *GBP1*, *NLRC5*, and *PSMB9*. On the other hand, *IRF1* is a member of the interferon regulatory transcription factor family. In agreement with previous reports that *GBP1* and *PSMB9* could be regulated by *IRF1* [91,92], this study revealed the promotor regions identified in *GBP1* and *PSMB9* contain the *IRF1* binding site. It is worth noting that *STAT1*, *IRF1*, *GBP1*, and *PSMB9* are IFN-gamma response genes, and *NLRC5* is a specific transactivator of MHC class I genes that are highly inducible by IFN-gamma stimulation [93]. Therefore, the upregulation of genes related to immune response processes in the CM layer on day 15 of pregnancy could be explained by a hypothesis that conceptus IFN-gamma might be able to activate the *cis*-regulatory elements that could interact with STAT1 and IRF1, which in turn upregulate the expression of the genes, such as *GBP1*, *NLRC5*, and *PSMB9*.

In conclusion, this study, for the first time, characterized the transcriptome and histone modification profiles for H3K4me3 and H3K27ac in pig uterine CM and LM layers during implantation. In addition, the distinct molecular features between the two layers were identified. Furthermore, the study identified the regulatory regions marked by H3K4me3 with transcriptional activity in the two layers.

## Figures and Tables

**Table 1:** Prediction of the binding sites of transcription factors within the TSS ± 1 kb region of immune response processes−related genes in GD15M_CM *.

DEGs	TFs	Binding Motifs	Binding Sites	Distance to TSS (bp)
IRF1	STAT1	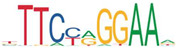	TTTCCAGTAAC	606
NLRC5	STAT1	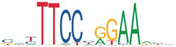	GATTTCCCGGCAGCG	252
GBP1	STAT1	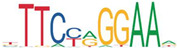	TTTCTAGGAAT	−20
	IRF1	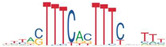	AAACACTTCCACTTTTGGTTT	72
PSMB9	STAT1	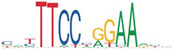	TTTATGGGAAA	−197
	IRF1	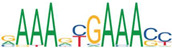	GAAAGGGAAAAC	259

* DEGs, differentially expressed genes; TFs, transcription factors.

## Data Availability

The RNA-seq and ChIP-seq data in this study were deposited into the NCBI (national center for biotechnology information) Sequence Read Archive database (PRJNA893617). The GEO accession of the datasets is GSE216440, and the link is https://www.ncbi.nlm.nih.gov/geo/query/acc.cgi?acc=GSE216440.

## Supplementary Materials

The following supporting information can be downloaded at: https://www.mdpi.com/article/10.3390/biom13010045/s1, Figure S1: Clustering and Venn analysis of the transcriptome data of the M_CM and M_LM on GD12 and GD15 in pigs; Figure S2: Clustering and principal component analysis of the transcriptome data of the M_CM and M_LM on GD15 and CD15 in pigs; Figure S3: Validation of the DEGs; Figure S4: The replicated IGV views of the H3K4me3 modification patterns; Table S1: Information of the RNA-seq data, ChIP-seq data, and primers.; Table S2: Information of the identified DEGs and GO terms.; Table S3: Information of the H3K4me3 and H3K27ac peaks identified in the M_CM and M_LM.; Table S4: Information of the H3K4me3 and H3K27ac differential peaks; Table S5: Information of the H3K4me3 and H3K27ac differential peaks within ±3 kb regions from the TSS of the DEGs.
