# Supplementary material for: Integrating Transcriptomic and ChIP-Seq Reveals Important Regulatory Regions Modulating Gene Expression in Myometrium during Implantation in Pigs
Source: Biomolecules. 2022 Dec 26;13(1):45. doi: 10.3390/biom13010045 (PMC9856092; doi:10.3390/biom13010045)
Supplement: Supplementary file 1 [file biomolecules-13-00045-s001.zip › Supplementary Files/Supplement Figures.pdf]

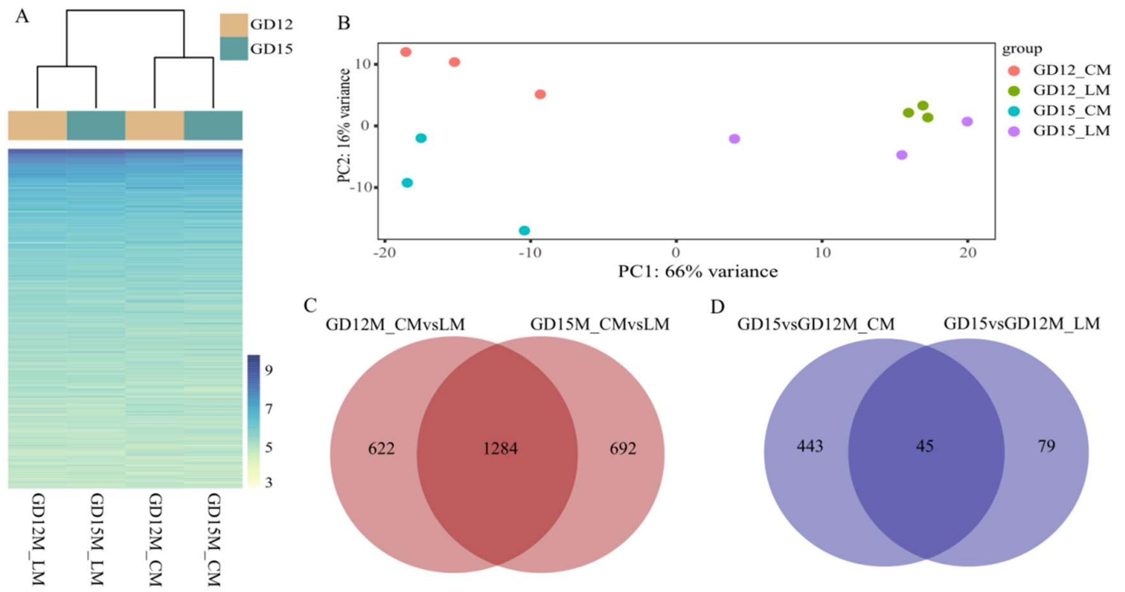

Figure S1. Clustering and Venn analysis of the transcriptome data of the M_CM and M_LM on GD12 and GD15 in pigs. (**A**) Hierarchical Clustering analysis of the transcriptome data of the M_CM and M_LM on GD12 and GD15. (**B**) Principal component analysis of the transcriptome data of the M_CM and M_LM on GD12 and GD15. (**C**) Venn analysis of the DEGs between M_CM and M_LM on GD12 and GD15. (**D**) Venn analysis of the DEGs in the M_CM and M_LM between GD12 and GD15. GD12, day 12 of pregnancy; GD15, day 15 of pregnancy; M, the mesometrial side of the uterus; CM, the circular muscle of myometrium; LM, the longitudinal muscle of myometrium; DEGs, differentially expressed genes.

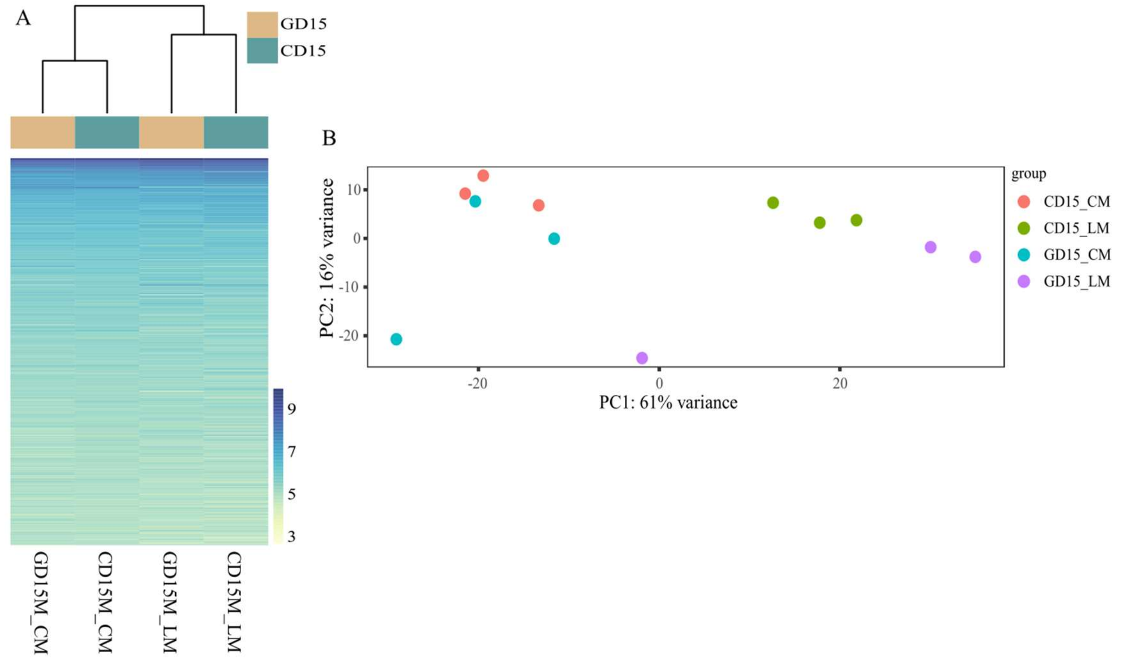

Figure S2. Clustering and principal component analysis of the transcriptome data of the M_CM and M_LM on GD15 and CD15 in pigs. (**A**) Hierarchical clustering analysis of the transcriptome data of the M_CM and M_LM on GD15 and CD15. (**B**) Principal component analysis of the transcriptome data of the M_CM and M_LM on GD15 and CD15. GD15, day 15 of pregnancy; CD15, day 15 of the estrous

cycle; M, the mesometrial side of the uterus; CM, the circular muscle of myometrium; LM, the longitudinal muscle of myometrium.

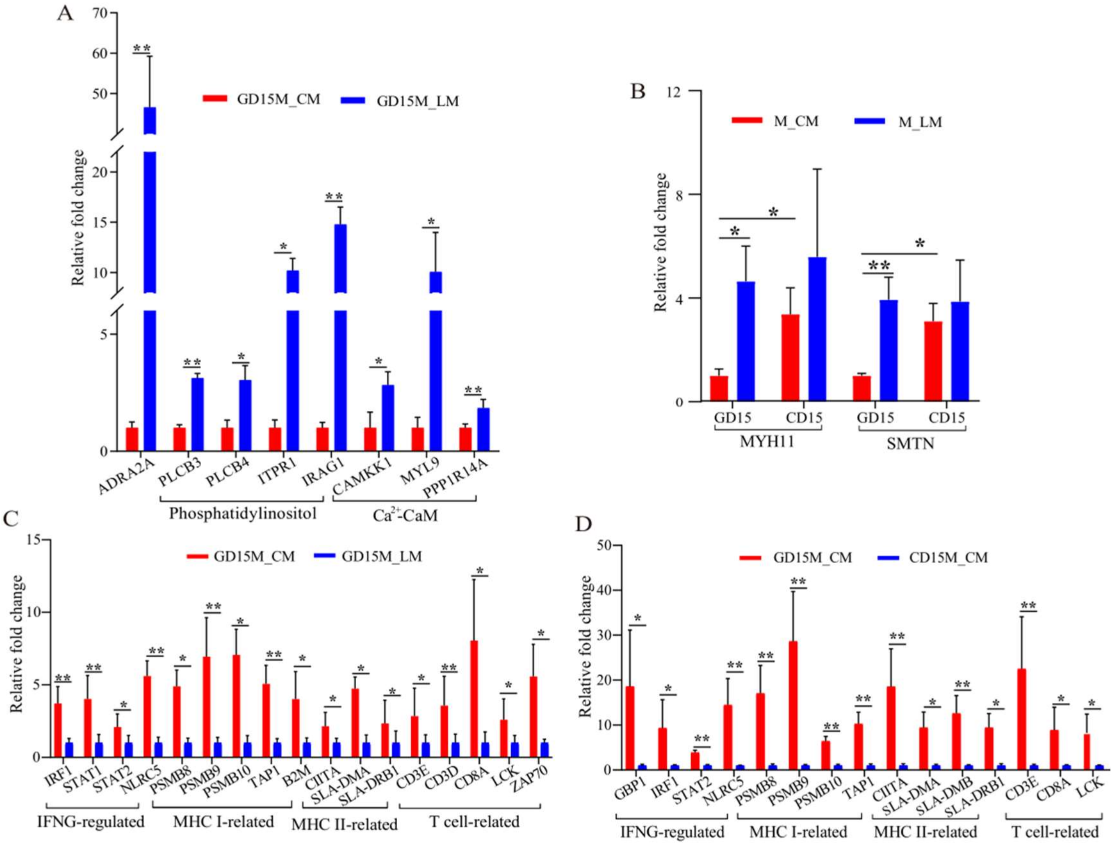

Figure S3. Validation of the DEGs. (**A**) Validation of the expression of the *ADRA2A* and some genes in the phosphatidylinositol and Ca^{2+}–CaM signaling pathways in M_CM and M_LM on GD15. (**B**) Validation of the expression of the two smooth muscle contraction marker genes (*MYH11* and *SMTN*) in M_CM and M_LM on GD15 and CD15. (**C**) Validation of the expression of the immune response processes–related genes in M_CM and M_LM on GD15. (**D**) Validation of the expression of the immune response processes–related genes in M_CM on GD15 and CD15. Data ($n \geq 3$) are represented as mean ± SEM, and * $P < 0.05$, ** $P < 0.01$. GD15, day 15 of pregnancy; CD15, day 15 of the estrous cycle; M, the mesometrial side of the uterus; CM, the circular muscle of myometrium; LM, the longitudinal muscle of myometrium; DEGs, differentially expressed genes.

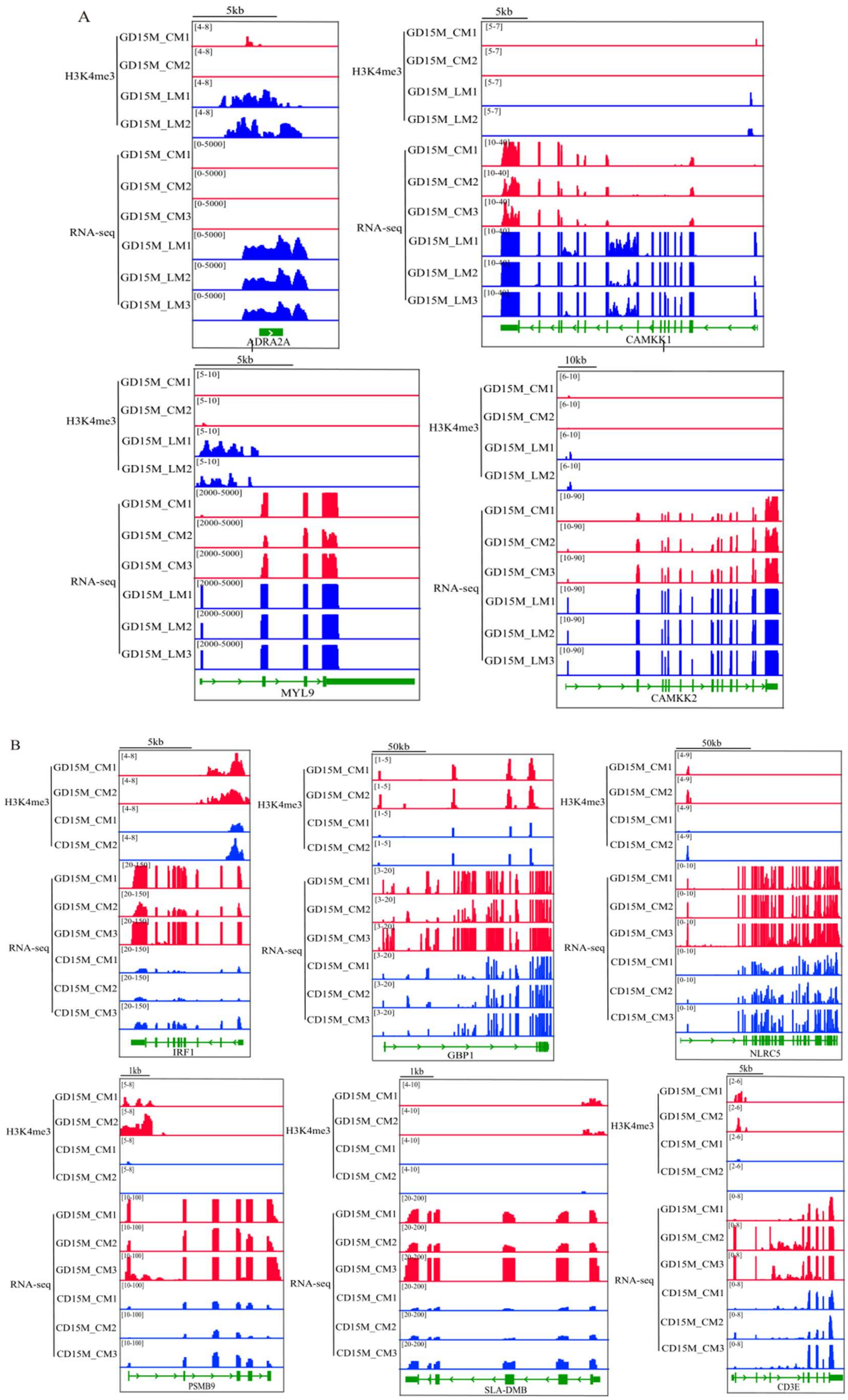

Figure S4. The replicated IGV views of the H3K4me3 modification patterns. (**A**) H3K4me3 modification

patterns of smooth muscle contraction−related genes. (**B**) H3K4me3 modification patterns of immune response processes−related genes. The peak calling of H3K4me3 and H3K27ac peaks in each replication of ChIP−seq data was performed by MACS2 (v2.2.6) with a 3 kb extension regions. The H3K4me3 and H3K27ac peaks were normalized with IgG files as control using bamCompare of deepTools (v3.5.1). GD15, day 15 of pregnancy; CD15, day 15 of the estrous cycle; M, the mesometrial side of the uterus; CM, the circular muscle of myometrium; LM, the longitudinal muscle of myometrium.
